# Cost–benefit analysis of installing dust control devices in the agate industry, Khambhat (Gujarat)

**DOI:** 10.4103/0019-5278.44694

**Published:** 2008-12

**Authors:** Lakho J. Bhagia, H. G. Sadhu

**Affiliations:** National Institute of Occupational Health, Meghani Nagar, Ahmedabad - 380 016, India

**Keywords:** Agate industry, cost–benefit analysis, silicosis

## Abstract

It is well known that an exposure to crystalline silica gives rise to silicosis and silico-tuberculosis (TB). In the agate industry of Khambhat (Gujarat) not only workers but also people staying in the vicinity of the agate-grinding facilities are exposed to crystalline silica. To reduce their dust exposure, dust control devices were developed. There are approximately 500 grinding machines located in Khambhat. A cost–benefit analysis of installing dust control devices on all agate-grinding machines was carried out by adding all positive factors and benefits and subtracting the negatives and costs. It was concluded that by installing dust control devices not only could the prevalence of silicosis and TB be reduced but also, in the long run, there could be financial benefits.

## INTRODUCTION

A cost–benefit analysis (CBA) finds, quantifies and adds all the positive factors. These are the benefits. Then, it identifies, quantifies and subtracts all the negatives, the costs. The difference between the two indicates whether the planned action is advisable. The real trick to doing a cost benefit analysis well is to make sure that you include all the costs and all the benefits and properly quantify them.

CBA is performed to determine how well, or how poorly, a planned action will turn out. Although a CBA can be used for almost anything, it is most commonly carried out on financial questions.

The process involves monetary value of initial and ongoing expenses vs. expected return. Constructing plausible measures of the costs and benefits of specific actions is often very difficult. In practice, analysts try to estimate costs and benefits by using survey methods.

The most common problem in the work environment of any industry is that the pollutant levels are generally higher than the permissible limits, barring some large-scale industries where proper care is taken to reduce occupational health hazards. In medium-and small-scale or cottage industries, there is poor or no engineering control.

A project entitled Prevention, control and treatment of silicosis and silico-tuberculosis in agate industry, sponsored by the ministry of heath and family welfare (Govt. of India) was completed at the National Institute of Health (NIOH) and the report was submitted to the ministry.[1] An attempt is made in this paper to do a CBA for installation of dust control devices in an unorganized agate industry in Khambhat (Gujarat).

The agate industry has been developed primarily as a cottage or household industry mainly located in Khambhat, Gujarat. This industry earns a valuable foreign exchange worth millions of rupees. Various articles like key chains, necklaces, art pieces, etc. are manufactured. The process of making such articles includes baking of stones, chipping, grinding, drilling and polishing. Of all these processes, grinding is the most hazardous.

There are two types of grinding machines viz. horizontal and vertical shaft grinding machines. The horizontal shaft machines are used to give only spherical shapes whereas machines with vertical shaft are used to give any shape to the stone.[1] There are approximately 500 grinding machines in Khambhat. A lot of dust containing crystalline silica is produced in the work environment of the grinding facilities[2] It is known that exposure to crystalline silica gives rise to a lung disease called silicosis.[3–6] Exposure to this type of dust produces not only silicosis but also predisposes to the development of tuberculosis (TB).[1] Recently,[7] it has been shown that crystalline silica (quartz) is a carcinogen also. Because the agate-grinding facilities are located in residential areas, not only workers but also family members and people residing in the vicinity are exposed.[8] This includes elderly sick people as well as children.

## METHODOLOGY

The agate-grinding facilities are situated mainly in Khambhat, with a population of 125,000. There are approximately 500 grinding machines in Khambhat. Approximately 20% of the Khambhat population stays in agate-dominated areas like Shakarpur, Vadva-Metpur, Bhoi-Bari, Hajju Fajju and Jumma Masjid areas.

There is no information available on the exact number of agate grinders, past grinders, the family or neighbors directly or indirectly exposed to the dust generated by the agate-grinding machines. Therefore, it was decided to prepare a list of persons by carrying out a door-to-door survey in Khambhat. Accordingly, a preliminary list of 5080 subjects was prepared. These were classified as present agate grinders (593), past agate grinders (533), family members (599), neighbors (1706) and other workers (1649) [Table 1]. The subjects of the category of present grinders were working in the process of agate grinding at the time of the study whereas the past grinders were not working in agate grinding presently but were working there at some time in the past. Thus, the subjects of these two categories had an occupational history suggesting direct exposure to the silica dust either in the present or in the past. The subjects under the categories neighbors and family had no occupational history involving silica dust exposure. The family category included those subjects residing in the houses where agate-grinding machines are situated. Similarly, the neighbors resided in the neighborhood of the houses having agate-grinding machines. The non-grinders were not engaged in agate grinding in the present or in the past but were working in other processes like stone heating, chipping, drilling, polishing, etc. As these processes do not generate much silica Dust, these workers are not exposed much to the silica dust during their work but are exposed to the silica dust generated by the agate-grinding machines located in the neighboring houses. Therefore, the neighbors, family and non-grinder categories are indirectly exposed to the silica dust. Of the 5080 subjects, a total of 1927 subjects were randomly selected for inclusion in the epidemiological study consisting of 397 present grinders, 341 past grinders, 127 family members, 748 neighbors and 314 non-grinders.

**Table 1:** Breakup of subjects

Category	No. of subjects	Percentage
Grinders	1126	20
Family members	599	12
Neighbors	1706	34
Non-grinders	1649	32
Total	5080	100

In 2004, the study included collection of baseline information such as prevalence of dust-related diseases and installation of dust control devices. After a period of 2 years, i.e., in 2006, the CBA was performed. The CBA was performed considering the number of grinding machines, cost of dust control devices, depreciation and maintenance of machines and the cost involved in the diagnosis and treatment of diseases.

## RESULTS AND DISCUSSION

### Prevalence of diseases

Table 1 shows the breakup of different categories of subjects registered in the door-to-door survey.

The prevalence of silicosis, silico-tuberculosis and TB among 1927 subjects is shown in Table 2. For convenience, the subjects were categorized as grinders and others. The others include non-grinders, family members and neighbors or residents in the vicinity of the agate industry. In addition to these subjects, 841 controls were selected from the villages Rohini and Pandad, 20 km from Khambhat. There is no industry of any type in these villages.

**Table 2:** Prevalence of diseases[1]

Category	Tuberculosis (%)	Silicosis (%)	Silico-tuberculosis (%)
Grinders	37	32	20
Others	20	8	4
Controls	4	0	0

Based on the prevalence of diseases in the study population, the number of people having diseases was estimated in Khambhat. The prevalence of TB in controls was found to be 4%.[1] This was subtracted from the cases of TB in Khambhat so that the remaining cases could be attributed to silica dust.

### Dust control device

To reduce the dust emission from grinding machines, a dust control device was designed and installed on both types of grinding machines. The dust control device includes a hood, flexible pipes and a suction motor of 0.5 HP and a bag filter specially designed for agate-grinding machines. The efficacy of the device in reducing dust exposure was found to be 93%.[1–2]

### Calculation of benefits

*Exposed population:* As mentioned earlier, all the positive factors are benefits. Installation of dust control devices will minimize the exposure to silica dust. In our study, the positive factors are saving the cost of diagnosis and treatment of people exposed to silica dust and their earnings if they do not get disabled. According to the census of India, the population of Khambhat was 80,439 in 2001. But, according to the locals, the approximate population of Khambhat in 2007 was 1.25 lakhs, of which about 20% of the population stays in agate-dominated areas like Shakarpur, Vadva-Metpur, Bhoi Bari, Jumma Masjid and Hajju Fajju. Hence,

People residing in agate-dominated area = 125,000*0.20 = 25,000

Number of grinders in Khambhat is about 20% as per Table 1. Hence,

No. of grinders in Khambat = 25,000*0.20 = 5000

The number of other persons exposed to silica dust in Khambat village (family members + neighbors + non-grinders) constitutes 80%of the exposed population. Hence,

Others = 25,000*0.80 = 20,000

### Number of TB, silicosis and silico-tuberculosis cases among grinders

The prevalence of TB among grinders is 37%. Hence,

No. of TB cases = 5000*0.37 = 1850

The prevalence of TB in controls is 4% [Table 2]. Hence,

No. of TB cases attributable to silica = 1850 – (0.04*5000) = 1650

The prevalence of silicosis among grinders is 32 %. Hence,

No. of cases of silicosis among grinders = 5000*0.32 = 1600

Prevalence of silico-tuberculosis in grinders = 5000*0.20 = 1000

(1) Total cases of TB, silicosis and silico-tuberculosis in grinders = 1650 + 1600 + 1000 = 4250

### Number of TB, silicosis and silico-tuberculosis cases among others

The prevalence of TB among the others category is 20%. Hence,

No. of TB cases in others = 20,000*0.2 = 4000

The prevalence of TB in controls is 4%. Hence,

No. of TB cases attributable to silica = 4000 – (20,000*0.04) = 3200

The prevalence of silicosis in the others category is 8%. Hence,

No. of persons having silicosis = 20,000*0.08 = 1600

The prevalence of silico-tuberculosis in others = 20,000*0.04 = 800

(2) The total cases of Tb, silicosis and silico-tuberculosis in the others category = 3200 + 1600 + 800 = 5600

### Total cases

(3) Total cases of diseases in Khambhat = (1) + (2) = 4250 + 5600 = 9850

Assuming that disease occurs in 10 years, we have estimated the cases of diseases per year.

No. of persons getting diseases per year is 9850/10 = 985

(4) Cost involved in treatment and diagnosis @ Rs.4000/- per case = 985*4000 = Rs. 3,940,000

In case of installing a dust control device on the machines, persons can work for many more years as people get diseases in their thirties and forties.

Assuming that the daily earnings of a person is Rs.80/- per day and there are 300 working days in a year, a person can earn Rs.24,000 per year. Hence,

(5) Total earning of silica-related cases per year = 500*24,000 = Rs. 12,000,000/-

In the above calculation of earnings, out of 985 cases per year the cases of TB (485) are not considered because TB is curable.

The total expenditure of diagnosis and treatment of diseases and their wages and/or productivity losses can be saved if installing dust control devices on grinding machines with vertical and horizontal shafts can minimize the exposure to silica dust. Therefore, the total benefit is the sum of (4) and (5).

(6) Total  benefit = Rs. 3,940,000/- + Rs.12,000,000 = Rs.15,940,000/-

### Maintenance of the dust control device

Most of the machines operate without a dust control device, with an electric consumption of 0.25 HP. We have designed a dust control device in which the emery wheel and the blower fan are derived with the motor, with an electric consumption of 0.5 HP. The extra electric consumption cost per day is Rs. 5/-. The dust collected in the bag filter is used for polishing. The cost of the dust collected is Rs.10/- per day. Thus, there is a net profit of Rs. 5/- per day.

Net profit due to sale of dust = 300*5 = Rs. 1500/- per year.

The cost of changing the bag filter 3 times a year is Rs. 1500/-.

The cost of repairing/rewinding the electric motor once a year = Rs. 200/-.

This is required for the grinding machines routinely even without using the dust control device.

Hence, the maintenance charges can be taken as zero.

### Calculation of costs

The cost of installing a dust control device is Rs. 8000/- per machine.

Depreciation of the dust control device @ 10% per year = 8000/10 = Rs. 800/-

(7) Total  depreciation  cost  of  500  machines = 500*800 = Rs.400,000/-

(8)(one-time investment) Cost of 500 dust control devices = 500*8000 = Rs.4,000,000/-

(9) Total cost = (7) + (8) = Rs.4,400,000/-

### Total savings

Total savings = total benefits – total costs

(10) Total savings for the first year = (6)-(9) = Rs.15,940,000 − Rs.4,400,000 = Rs. 11,540,000/-

Because the cost of installing dust control devices on machines is only once,

Total savings for subsequent years = (10) - (7) = Rs. 15,940,000 – Rs. 400,000 = Rs. 15,540,000/-

Thus, CBA shows that every year millions of rupees can be saved by controlling silica dust in the agate industry. The benefits may be much more than that depicted here if the cost of research projects on occupational health in the agate industry by national institutes and non-government organizations is also considered.

## CONCLUSIONS

CBA shows that if dust control devices are installed in all the agate-grinding facilities of Khambhat, not only the prevalence of Silicosis and TB can be reduced, but in the long run there are financial benefits also.

Agate is an unorganized industry. Grinding of stones is carried at the places of residence. Because the cost of diagnosis and treatment is borne mostly by government hospitals and institutes, owners/workers do not wish to invest Rs. 8000/- on dust control devices. But, looking at the benefits of the control of dust exposure, the government and the association of agate manufacturers should come together to solve the problem. Moreover, people residing in the vicinity of the grinding facilities are also exposed to silica dust and hence necessary steps should be taken urgently to modernize the agate industry.

## References

[1] Prevention, control and treatment of silicosis and silico tuberculosis in agate industry- report by submitted by NIOH to the Ministry of Health and family welfare, Govt. of India, 2004.

[2] Bhagia LJ, Parikh DJ, Pandya GL, Vyas JB, Saiyed HN (2007). Assessment and control of silica dust exposure in agate grinding units, Khambhat, India. Indian J Occup Hyg Safety.

[3] Saiyed HN, Ghodasara NB, Sathwara NG, Patel GC, Parikh DJ, Kashyap SK (1995). Dustiness, Silicosis and tuberculosis in small scale pottery workers. Indian J Med Res.

[4] Saiyed HN, Parikh DJ, Ghodasara NB, Sharma YK, Patel GC, Chatterjee SK (1985). Silicosis in slate pencil workers: An environmental/medical study. Am J Ind Med.

[5] Saiyed HN, Chatterjee BB (1985). Rapid progression of silicosis in slate pencil workers: A follow up study. Am J Ind Med.

[6] Sadhu HG, Parikh DJ, Sharma YK, Saiyed HN, Rao PV, Kulkarni PK (1995). A follow up study of health status of small-scale agate industry workers. Indian J Ind Med.

[7] (1997). IARC monographs on the evaluation of carcinogenic risks to humans: Silica, some silicates, coal dust and para-ararnid fibrils.

[8] Bhagia LJ, Parikh DJ, Saiyed HN (2007). Ambient silica monitoring in vicinity of agate industry, Khambhat, India. Indian J Occup Hyg Safety.

